# Identifying the experience of geographical narcissism during medical education and training

**DOI:** 10.1007/s10459-025-10440-9

**Published:** 2025-05-10

**Authors:** Riitta Partanen, Diann Eley, Remo Ostini, Matthew McGrail

**Affiliations:** 1 Rural Clinical School, The University of Queensland, Hervey Bay, Australia; 2 Medical School, The University of Queensland, Brisbane, Australia; 3 Rural Clinical School, The University of Queensland, Toowoomba, Australia; 4 Rural Clinical School, Australian National University, Canberra, Australia; 5 Rural Clinical School, The University of Queensland, Rockhampton, Australia

**Keywords:** Education, Medical, Rural health, Healthcare disparities, Geographical narcissism, Career decision making

## Abstract

Inequality of healthcare provision between metropolitan and rural communities is an enduring international concern. Geographical narcissism (GN) in medicine is a belief that the best healthcare provision only occurs in the big cities. In healthcare education and workforce development, there is a growing sentiment that GN exists, and is likely contributing to access inequalities and rural workforce shortages. This qualitative study aimed to explore if and understand how, where, when and why GN was experienced by medical students and prevocational doctors during their medical education and training. A constructivism research paradigm was applied, and reflexive thematic analysis was utilised for inductive coding of the different experiences and perceptions of GN. Semi-structured interviews with 29 Australian medical students and prevocational doctors were undertaken. Four key themes, with ten subthemes, were identified. First, GN is part of the hidden curriculum of medical education and training. Second, GN occurs across the continuum of medical education and training. Third, GN occurs with medical career advice. Fourth, GN is demonstrated by models of healthcare and health systems. This exploratory study confirmed previous anecdotal evidence that GN exists in healthcare and has outlined how, where, when and why it is experienced during medical education and training. Ignoring the potential damage and consequences of GN perpetuates the established metrocentric models of medical education, training, workplace culture, healthcare service provision and investment. The cycle of inequality will persist for rural populations, including poorer health outcomes and the rural medical workforce shortfall will continue – in part due to GN.

**Supplementary Information:**

The online version contains supplementary material available at 10.1007/s10459-025-10440-9.

## Introduction

Rural communities worldwide continue to have inadequate access to the healthcare they need close to home. The ongoing rural medical and other healthcare professional workforce shortages result in health inequalities between metropolitan and rural populations (Baazeem et al., 2024; Edelman et al., 2020; Kavanagh et al., 2023; Keeves et al., 2021; Rolfe et al., 2017). Despite the growing evidence base of policies, strategies and initiatives that improve rural medical education, training, and workforce, most countries are yet to achieve an equitable distribution of the medical workforce (World Health Organization, 2019, 2021).

Decades of research have focused on tangible interventions such as increased selection of rural background students, expanded rural medical education and training and return of service obligations (Henry et al., 2009; Kwan et al., 2017; McGrail et al., 2023; O’Sullivan et al., 2021, 2025; Playford et al., 2017). Additionally, partner and family considerations, lifestyle goals and perceived career opportunities (Cano et al., 2021; Eley et al., 2012; Laurence & Elliott, 2007; Rogers et al., 2010) as well as personality traits (Campbell et al., 2016; Eley et al., 2019) are better understood regarding their contribution to career decisions in medicine. However there has been little research into whether there are specific experiences during either rural or urban based medical education and training that influence future workplace location. Are there critical, preventable experiences that deter those with an interest in a rural medical career from choosing this path? This is a pivotal time in a young doctor’s career where experiences during training can be a powerful factor not only in deciding a speciality to pursue but also their long-term practice location. Experiencing negative messages, viewpoints or direct comments about choosing a rural medical career can have a strong and lasting impression on students and trainees who are vulnerable to influences on their decisions. Supervisors and educators that portray rural medicine in a negative context are likely enacting what is known as geographical narcissism (Fors, 2018).

Geographical narcissism (GN) is the subtle, often unconscious devaluation of non-metropolitan (‘rural’) knowledge and expertise, stemming from the perception that metropolitan knowledge and expertise is the standard or superior (Fors, 2018). It is not just linked to health. Cultural, social, and economic influences have contributed to the GN phenomenon, impacting society’s functioning, wealth, health, and overall well-being. Throughout history, large cities in metropolitan areas have been traditionally viewed in a hierarchical manner, with power, leadership, decision-making, and significance usually emanating from these centres (Wilkinson et al., 2022, 2023; Winther, 2014).

GN is believed to parallel other prejudices, such as sexism, racism, ageism, and has a similar continuum, with a large and highly urbanised community at one end of the spectrum and a very small and remote community at the other (Fors, 2024). The farther the rural setting from a metropolitan centre, the lower its perceived status, influence, opportunities and quality of life compared to the higher status, power, and privileges typically associated with metropolitan settings (Pun-Cheng, 2016). Those who have only lived in a metropolitan setting will often not appreciate or recognise their own metropolitan privilege stemming from GN (Fors, 2018). Until the GN term was coined within published literature in 2018 (Fors, 2018) most of the narrative relating to the societal implication of different geographies referred to this more simply as the metropolitan-rural divide (Huning et al., 2012). Subsequently, the GN phenomenon has been recognised across different facets of society and careers with rural people coming across the negative stereotyping, deficit discourse and lack of respect regarding living and working rurally from some metropolitan colleagues, friends or relatives (Baker, 2019; Davis, 2021; Diprose, 2019; Neilson, 2019). In medicine, rural locations are sometimes seen as suitable for combining vacations to get away from the hectic pace of the city and provide additional healthcare through outreach services. However, while for some metropolitan clinicians the decision to provide this regular visiting service is altruistic, for the majority their primary motivation is to grow their own professional careers (O’Sullivan et al., 2017). This rural exploitation is an example of GN, which further marginalises those who live and work in these communities year-round.

In the field of healthcare education and workforce development, there is a growing evidence base suggesting that GN exists and likely impacts on the health outcomes of rural people, but to date published evidence of specific GN experiences is lacking (Hayes et al., 2025; Malatzky et al., 2020; Probst et al., 2019; Roberts et al., 2021). The potential impact of GN in medicine may be more prominent, considering specialist training continues long after they join the workforce. It is therefore important to understand how GN is experienced by medical students and recent graduate (prevocational) doctors, during medical school and in the workplace. Set in Australia, this study aimed to understand how geographical narcissism was experienced (e.g., who, where, when and why) by medical students and prevocational doctors during their medical education and training.

## Methods

To effectively understand how GN was experienced, this study required a subjective inductive approach to the research (Varpio et al., 2020). This bottom-up approach enabled the researchers to understand and identify patterns of the GN phenomenon during medical education and training. With reality being socially and experientially constructed, to understand the reality of GN, the constructivism research paradigm with a qualitative exploratory approach was utilised. Constructivism’s inductive approach facilitated the exploration of diversity for each individual’s experiences and perceptions of GN, recognising that different people may perceive multiple realities (Kumar, 2014; Liamputtong, 2019).

### Setting and participants

Medical students in the final two years of their 4-year Doctor of Medicine program and based at either a metropolitan or rural clinical education unit were recruited from one Australian medical school based in Queensland. The decision to only invite students from the one university was because the cohort of students in both rural and metropolitan settings was large enough and the geographical area of the university’s footprint is almost 1 million square kilometres, equating to an area nearly twice as large as Spain.

The prevocational doctors were recruited from two large rural hospitals and one tertiary metropolitan hospital in Queensland, Australia. In Australia, until a medical graduate commences their specialty training, they are referred to as prevocational doctors. Generally, most doctors commence vocational (specialty) training somewhere between three and six years following medical school graduation. The decision to limit the prevocational doctor locations was in part due to using the convenience method of sampling and a pragmatic one to limit the complexity of requiring research agreements with each site while still having large enough cohorts to recruit the participants.

The definition of rural in this study referred to any community with a population of less than 200 000 inhabitants. The term rural was chosen because in Australia most medical schools with training based in regional, rural and/or remote locations are collectively called Rural Clinical Schools, and one of the locations has a population of almost 200,000.

### Recruitment

All medical students and prevocational doctors from the above-mentioned cohorts were invited to participate. Participants for both cohorts were recruited by email, articles in electronic newsletters, flyers placed in common areas, social media posts and via word of mouth. Snowballing from participants was also encouraged, making use of participant networks.

### Consent

We obtained informed consent from each participant, after they had reviewed the participant information sheet which was emailed to them. Participants who then emailed advising they wished to participate, implied consent. Consent for publication was not applicable as no identifying data were collected or published.

### Data collection

Interviews were semi-structured, which used a series of questions exploring the participant’s experiences with rural medical education and/or training, future career and workplace interest and their experiences of GN during their medical education and additionally for the prevocational doctors during their working career so far (Interview guides – Appendix). Whilst the pre-interview participant information sheet included GN in the title and aim of the project, the topic of GN was only explicitly brought in towards the latter part of the interview when participants were asked to reflect on their experiences of GN, after being provided a full definition. Online videoconferencing was used for all interviews and were conducted by the principal researcher (RP), recorded and transcribed verbatim. Limited demographic data were collected including age, gender, childhood, education and training geographic locations and year level in medicine or post-graduate year. Each participant was allocated a unique code using consecutive numbers within each group. This ensured anonymity, as well as the ability to remove their data if requested. Participants received a small honorarium in appreciation of their time.

### Data analysis

Reflexive thematic analysis (RTA) was used for the data analysis, facilitating the deep interpretation of the rich and complex data to determine the significance and meaning of the patterns identified from the participants’ GN experiences. (Braun et al., 2016, 2019; Braun & Clarke, 2006). By choosing RTA, the researchers’ own perspectives, values, and knowledge regarding rural medical education and training and GN, were acknowledged but also embraced. However, to ensure a robust yet fluid analysis, the RTA process required reflexive, iterative and reiterative engagement with the dataset so that unintended assumptions about GN were not embedded in the analyses. (Braun & Clarke, 2019; Clarke, 2018; Olmos-Vega et al., 2023).

Initial codes were created by the interviewer and first author a highly experienced rural medical educator and general practitioner (RP). These codes initially aligned with the interview questions, with each interview subsequently reviewed twice resulting in the refinement and modification of codes. An experienced medical workforce researcher assisted in verifying the themes and accuracy of intent. (MM). Initially the codes were semantic, representing what the participants were saying. Subsequent codes were latent adding in assumptions of the underlying meaning including the social and cultural construct of the participants. During the coding and recoding process, discussions occurred between fellow researchers all of whom have substantial working experience and research insights of medical education across all contexts (RP, DE, RO, MM). NVivo (Version 14) was used to assist in data management, analysis and coding process.

Following the coding process, themes were generated to help identify how, where, when and why GN was experienced during medical education and training. The defining and naming of themes and subthemes evolved with repeated reflection and revision of the codes, quotes and dataset.

### Reflexivity

Reflexivity in qualitative research, requires researchers to consistently reflect on how their personal background, experiences, and assumptions may shape data analysis and the findings (Olmos-Vega et al., 2023).All four researchers have extensive rural experiences related to either growing up or working in rural communities.

The first author (RP) grew up on a farm in a small rural town, is a highly experienced medical educator and general practitioner, with over 30 years working and residing in rural communities. Her extensive rural clinical and medical education background provided rich insights which had been shaped by personal experiences of rural prejudice. The second author (DE) grew up on a farm in the USA. After moving to Australia her research focus was on rural health workforce both in Australia and the USA. She maintains a strong interest in rural workforce the essence of which is the shortage of medical training and facilities in rural locations worldwide. The third author (RO) also grew up on a small farm and experienced rural healthcare firsthand. This experience informed the direction of his health research work, which brings a behavioural science perspective to questions of health workforce decision-making. His medical ethics training and previous health literacy research have brought an equity focus to his current health research. This is the perspective he brings to understanding the geographical narcissism results reported in this manuscript. The final author (MM) grew up in metropolitan Melbourne, Australia, but his working career has wholly been in rural communities. His research focus is largely centred on improving access to healthcare for rural populations, which includes evidence supporting developing a medical workforce to meet the health needs of rural communities.

All authors acknowledged and reflected on their subjectivity and were committed to amplify the voices of the participants, ensuring the analysis captured a wide range of perspectives rather than reinforcing their own pre-existing narratives.

### Ethics approval

Ethics approvals were received from Royal Brisbane Women’s Hospital Human Research Ethics Committee (HREC/2021/QRBW/76335) and ratified by The University of Queensland Ethics and Integrity Committee (2021/HE001947).

## Results

### Demographics

Twenty-nine participants were interviewed over a 12-month period during 2021 and 2022. The interviews were between 40 and 60 min in duration. The participants included 15 medical students (MS) and 14 prevocational doctors (PVD), with a spread of demographic characteristics (Table 1). Most participants had both rural and metropolitan experiences during medical education and training. The participants’ demographic profile was representative of the wider domestic medical student and prevocational doctor population.

**Table 1:** Demographic profile of participants

	Categories	Medical students	Prevocational doctors
Gender	Female Male	6 9	6 8
Age	21–25 26–30 31+	8 4 3	5 6 3
Childhood background	Rural Metropolitan	6 9	5 9
Current Doctor of Medicine year level	Year 3 Year 4	7 8	^a^NA
Medical School clinical years location	Rural only Metropolitan only Both Rural and Metropolitan	10 2 3	5 7 2
Medical degree location	Australia International	15 0	12 2
Current Prevocational doctor year	^b^ PGY 1 PGY 2 PGY 3–5	NA	6 6 2
Prevocational doctor workplace locations	Rural only Metropolitan only Both Rural and Metropolitan	NA	5 6 3

^a^NA – Not applicable; ^b^PGY – post-graduate year

### Themes

**Fig. 1:**
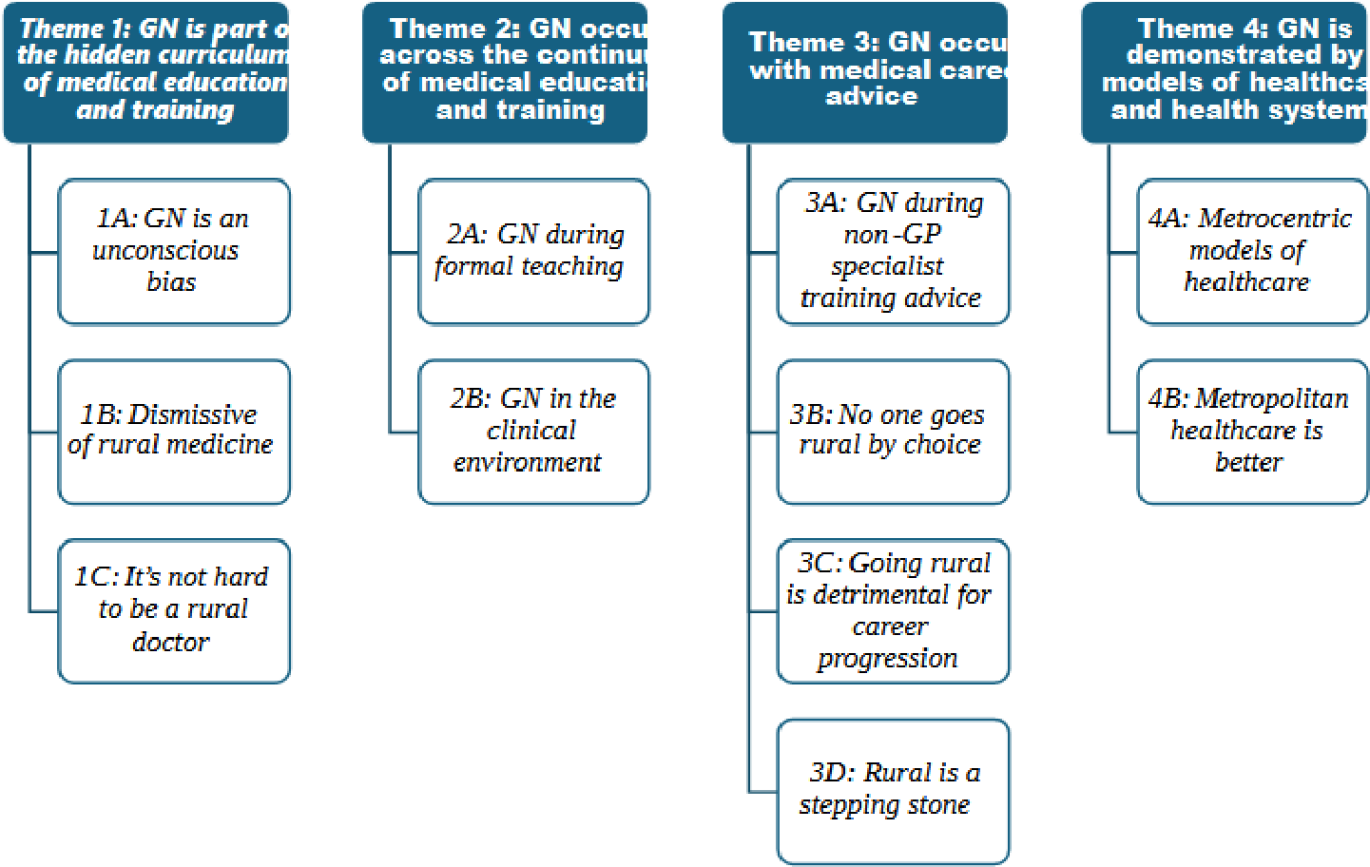
Thematic framework of key themes and subthemes

Four key themes and 11 subthemes were identified to describe how GN had been experienced by medical students and prevocational doctors during their medical education and training (Fig. 1). The themes relate to how, where, when and why GN occurs.

## Theme 1: GN is part of the hidden curriculum of medical education and training

Participants described GN as something they generally experienced covertly more often than overtly, suggesting it is part of the hidden curriculum and akin to other unconscious biases.

### 1A: GN is an unconscious bias

Participants expressed an underlying perception that rural medicine was presented and discussed as being inferior which was embedded within both the educational and workplace culture, which was thought to often not be a conscious decision by teachers or clinicians. GN is an unconscious bias against rurality which can also be called rural prejudice. This perception unwittingly permeated throughout the medical education and training continuum.

*I don’t know if I’ve not been the recipient of GN…But I think … it’s just sort of that culture…of that condescension…. As with all forms of discrimination*,* it’s often more subconscious than we would like it to be and it’s more the offhand comments rather than directed. (PVD6)*

*…when so much of the coursework is taught by people who don’t practice rurally*,* those sorts of undertones sort of more generalised (metro) elitism*,* is kind of how I interpret it. (MS12)*

*I think that there is a lot of belief (bias) that there is inferior care that occurs in rural areas as opposed to the perfection of care that occurs in the ivory towers of your (city). (MS3)*

*Unconsciously, I think we all label the best hospitals as the metropolitan hospitals. (PVD1)*

### 1B: Dismissive of rural medicine

Participants reported that rural medicine in healthcare was occasionally dismissed by educators or not discussed, rather than being overtly negative about it. The lack of acknowledgement of rural medicine, its relevance, the expertise, and the strengths of rural medicine, portrayed a message that rural medicine was not important. Participants noted that this message often came from those educators or clinicians with limited or no real-life experience in rural medical practice.

*People might be a little bit dismissive towards it (rural medicine)*,* but not … necessarily disparaging or negative. … but just apathetic about rural. (MS9)*

*The people who I felt were being the most dismissive about rural medicine were the ones who had the least exposure to it. (PVD13)*

### 1C: Not hard to become a rural Doctor

Participants reflected on the assumption that rural doctor recruitment was less competitive due to the chronic medical workforce shortages, which implied that working in a rural area required a lower level of skill than working in a metropolitan area. For international medical graduates (IMG), working in rural areas was sometimes considered a steppingstone to metropolitan medical practice, based on the assumption that they would not choose to stay longer than necessary.

*They’ll (metropolitan doctors) be like*,* the job rurally is really easy. I think it more just highlights that they have no idea of what rural practice often is. (MS11)*

*Because of the relative deficit of doctors out there*,* the barrier for entry is lower and that could be causing someone who might not have been as good of a clinician to go out there. (PVD4)*

## Theme 2: GN occurs across the continuum of medical education and training

Participants reported experiencing GN overtly during formal teaching and within the clinical environment predominantly in the metropolitan setting.

### 2A: GN during formal teaching

Learning topics during medical school sometimes had scenarios based in the rural context. Participants reported these were usually presented or discussed with a deficit lens and reinforcing the inferiority of rural medicine.

*I think in Case Based Learning (CBL) cases*,* that have been set in rural areas*,* the discussion starters have been around what services can’t you do rurally and is not really matched with any kind of positive lens or version of it about what can be offered rurally*,* or what are the benefits…. (MS12)*

*The lens was always what’s the limitations of being rural. What do they not have? They don’t have a CT scanner*,* so what are you going to do? They don’t have the same number of staff*,* so what are you going to do? (MS14)*

Participants identified that GN was demonstrated by other learning activities where topics were presented by “experts” in the field. These experts were usually metropolitan tertiary hospital based sub-specialists, who presented the cases based on a well-resourced metrocentric healthcare provision lens, inferring what high-quality healthcare is. Participants recognised that sub-specialists had a wealth of knowledge, but who only understood the metropolitan perspective around the topic, in itself was an example of GN.

, *… a very large amount of the education that we receive is from specialists*,* not generalists. All of their opinions through all of our lectures*,* all the way through*,* are tainted with the idea that you need to be a super specialist in order to provide the best care*,* and the super specialists are not outside of metropolitan areas. If you want to learn from the best*,* you’ve got to be near the best*,* which means you’ve got to be in (city). (MS10)*

### 2B: GN in the clinical environment

Participants recalled occasions when metropolitan clinicians were critical of rural training and rural medicine.

*When it (rural medicine) is talked about it*,* it’s talked about it as its own little subspecialty of medicine or it’s…. a little bit backwards. (MS8)*

*I do remember a surgeon in (metropolitan hospital) saying when I told him that I was going to go to (large rural hospital) to do an internship*,* the first thing he told me was don’t pick up bad habits.… (It) set up my expectation. (PVD2)*

Often this criticism of rural medicine and rural doctors occurred when patients were transferred to a tertiary metropolitan hospital for emergency and/or complex conditions, with limited recognition or understanding by the critical metropolitan doctor, of the rural context including resources and scope of practice.

*When they’re referred and asked for a takeover of care in (tertiary metropolitan hospital) … there’s that mismatch in expectations*,* when the city doctors think that the rural doctor should be able to take care of something*,* that’s where there’s some negative sentiment. (PVD11)*

*I don’t know if it was just (tertiary hospital)*,* but a lot of the doctors (say) this person was transferred from (rural)*,* and they didn’t do the right blood tests or didn’t put the right drain in … at the time I didn’t really think anything of it*,* but now I’m like*,* well*,* actually they probably just didn’t have the exact right materials that you have here. (MS13)*

## Theme 3: GN occurs with medical career advice

Medical students and prevocational doctors often seek career advice from those who have gone before them, as they plan their own medical career trajectories. Having navigated the training pathways and entry requirements and processes, vocational specialty trainees are quite influential for those who are still deciding their career path or waiting to join their preferred career path. During these conversations GN can be present. In Australia vocational specialty training is generally categorised as General Practice (e.g. Family Medicine and Rural Generalism) training or non-GP specialty (e.g. Internal Medicine, Surgery, Psychiatry) training.

### 3A: GN during non-GP specialist training advice

Participants planning a non-GP specialty career received advice indicating the need to spend their prevocational doctor years at a metropolitan hospital for two main reasons. One, higher quality training and two, to assist them in gaining entry onto their preferred training pathway.

*A couple of consultants when asking me about future career choices … make offhanded comments…. you won’t get far in your career if you are being taught at anywhere that’s not the (tertiary hospital) (MS7)*.

*I do remember … consultants who implied that it’s always better to get into a metro hospital*,* because it has a better progression … as compared to a (large rural) hospital. (PVD1)*

*… getting career advice (from many people) … recommending training or spending junior years at the major centres. Just that kind of advice and looking at people who I’ve seen succeed*,* looking at their career trajectory*,* it’s most often just been through those metropolitan areas.…. just what I’ve been exposed to. (PVD14)*

### 3B: No one goes rural by choice

Participants who had an interest in rural education, training or future practice recalled that they had been critically questioned about their rural intent. They reported a pervasive assumption by metropolitan doctors, that rural doctors were not there by choice, reinforcing the stigma around rural doctors.

*It’s always just that automatic assumption that if you’re in … a rural site you’ve been shafted as opposed to actually wanting to go there - which is quite sad because it’s actually way better. (MS13)*

*It’s very much that attitude…. doctors who work (in a large rural location) are working (there) because they couldn’t get a job in metro. (PVD13)*

### 3C: Going rural is detrimental for career progression

Participants were aware they had acquired the perception that going to a rural hospital for internship or subsequent post graduate years might be detrimental for their career progression in their preferred specialty.

*I have the impression that if you want to do surgery*,* you can’t do an internship in (large rural town)*,*…I don’t know why I hold that belief. But that seems to be filtered down to me from somewhere*,* different conversations I’ve had with people. (MS15)*

*Most people encourage you to do a rural term*,* but … do a shorter period of time (PVD14).*

### 3D: Rural is a stepping stone

Participants recalled receiving advice from consultants that going rural was useful for their learning, but with the caveat that it should only be temporary. The advice generally recommended that once they had gained the relevant valuable experience from the rural setting, they should return to metropolitan hospitals to fulfil their training requirements and work there in the longer term.

*Most (consultants) encourage you to do a rural term*,* but I think it’s generally always speaking in terms of do a shorter period of time. (PVD14)*

*I think the general attitude is that (large rural) is the way to step up into the city or a bigger placement. (PVD2)*

## Theme 4: GN is demonstrated by models of healthcare and health systems

Participants identified GN in the hierarchy and models of healthcare education, healthcare, health systems and the allocation of resources.

*I think it’s (GN) inferred from the way that these*,* (healthcare) organisations are set up. Even the way*,* university is set up. (MS11)*

### 4A: Metrocentric models of healthcare

Participants identified GN in the structure of the healthcare system and distribution of healthcare services across a jurisdiction. The metrocentric model of healthcare, with services being only available in large city hospitals rather than considering a geographical distribution based on incidence of presentations to determine where healthcare investment should be prioritised.

*We’ve shifted too far towards flying everyone in and out of the cities and delivering healthcare there*,* rather than remaining in place and allowing generalists to safely deliver high quality healthcare. (PVJD6)*

*Despite the fact that (large rural town) is the young stroke capital*,* we don’t have any clot retrieval services.…. It just kind of serves to underline the fact that*,* it’s not fair or egalitarian*,* it is the reality that we have. (MS10)*

Participants in the study recognised that patient travel subsidies, to access specialty services, were inadequate and disrespectful, as rural patients were still disadvantaged in accessing healthcare only available in the city. The funding provided generally does not cover the total cost of expenses accrued to attend the medical appointment, nor acknowledges the other indirect costs associated with needing to travel, such as carer responsibilities, time off work and loss of productivity.

*The travel subsidy that you get through hospitals if a certain specialty is not offered in*,* (rural town)*,* They’re like*,* don’t worry*,* we offer a travel subsidy to (large rural town or city). It might be $150 for fuel*,* but it’s ignorant of the fact that it’s three hours either side. You’ll probably most likely have to stay overnight unless they can get a midday appointment. All those things*,* like you having to take a day off work as well. It’s not equitable at all. (MS15)*

### 4B: Metropolitan healthcare is better

Participants heard from both patients and clinicians that healthcare must be better in a metropolitan location because it is the city. This perception relates to the investment in resources to manage complex cases and the less common conditions requiring sub-specialist care and may be the only location for certain interventions.

*I think there is a perception that you get better care in a tertiary centre*,* which is probably fair enough*,* because the resources are better. (PVD8)*

*… a bit of GN as well from patients themselves where they think that the bigger hospitals in the metro areas are going to give them better care than (large rural)*,* but really*,* it’s actually the same. (PVD13)*

Other participants who experienced working in a rural location reflected how metropolitan doctors often have inadequate understanding of the available resources (equipment and personnel) rurally which informs their decision of whether a patient needs to be transferred or not. The personnel not only relate to the doctor(s) or specialist skill(s) required, but also the other ancillary (nursing and allied health) healthcare staff required.

*I think people don’t understand necessarily that we’re not saying we can’t do something just because we don’t feel like doing it*,* but it’s because we physically can’t organise (the procedure)*,*…. So*,* I think limitations like that*,* people misinterpret. (PVD7)*

*In orthopaedics*,* if…*,* they don’t feel like they have the (multi-disciplinary) support. Sometimes the city doctors don’t understand the situation … That’s the reason for the negative sentiment. (PVD11)*

## Discussion

This is the first study to explore and identify specific experiences of GN during medical education and training, verifying its existence across the continuum, and through a variety of contexts, giving credibility to the GN narrative in society (Baker, 2019; Davis, 2021; Diprose, 2019; Neilson, 2019) and in the healthcare literature thus far (Fors, 2018, 2023; Probst et al., 2019). It confirms that GN represents a critical challenge within medical education and training, contributing to the systemic bias that favors metropolitan over rural clinical experiences, resourcing, and investment in healthcare, and is likely to be contributing to the ongoing medical workforce shortages in many rural areas globally.

A key finding was that GN manifested as an element of the hidden curriculum, similar to other attitudes and behaviours that operate within learning environments that are not explicitly part of the formal curriculum (Hafferty & Franks, 1994). These unintentional GN lessons are learned implicitly by example and/or as part of the culture of educational settings, healthcare institutions and health systems that do not consider rural medicine on an equal footing to metropolitan medicine. The GN bias facilitates the pervasive and prevailing narrative suggesting that prestige and competency in the medical field are closely tied to learning and practicing in metropolitan environments (Cano et al., 2021; Eley et al., 2012).

Accredited medical programs in Australia require the inclusion of rural medical clinical experiences, (Australian Medical Council, 2023); however, this study illuminates that GN can still permeate through the formal and informal medical curriculum, despite increased awareness and intent for positive promotion of rural medical education, training and careers. Models used for medical education, training and healthcare delivery are traditionally developed in metropolitan settings, with metropolitan resources and expertise factored into the designs. Such models exemplify GN when endorsed or accredited by metropolitan based teams of experts, including their direct application in rural settings with minimal flexibility to adapt to the rural setting and their differences of physical or human resources.

Most healthcare settings in which learning and training occurs support clinical guidelines and ways of working that are generally based on evidence from metropolitan based research studies (Knight et al., 2015; O’Sullivan et al., 2020; Wong Shee et al., 2022). These guidelines are therefore not evidence based or nuanced for the rural setting where decisions to deviate from a guideline are sometimes required. This deviation may inappropriately be perceived as poor healthcare or incompetence by metropolitan clinicians who lack understanding of the context in which the healthcare is being provided. Furthermore, each rural community and its resources differs between locations, noting the requirement for specific rural contextualisation (Chater, 2008).

Professional identity formation occurs during these formative years of medical school and prevocational training. This includes attitudes towards and interest in practicing rural medicine. Recurrent GN exposure risks perpetuating GN in the next generation of learners (Sternszus et al., 2024). Whilst few study participants acknowledged a direct impact from their GN experiences to date, such as reducing or removing their interest in working rurally, the range of identified themes confirms regular GN exposures across their learning. GN is a likely contributing factor for medical career preference changes away from rural, which are regularly observed elsewhere (Cano et al., 2021; O’Sullivan et al., 2019; Paynter & O’Sullivan, 2020; Rogers et al., 2010) and is likely to be undermining the well intentioned and evidence based policies currently in place to increase rural medical workforce numbers (McGrail et al., 2023).

Within the realm of medical career advice, GN is evident when aspirants were steered toward prestigious metropolitan tertiary institutions under the assumption that such training settings guarantee superior outcomes. This belief perpetuates the idea that high quality training and the “best” doctors emerge from metropolitan settings, overlooking the vital contributions and unique skill sets cultivated in rural practice (Cano et al., 2021). The narrative surrounding career progression often fails to acknowledge the complexities of medical training, where competencies can be developed in diverse environments. Rural healthcare practitioners are far more likely to understand the intersectionality that exists for their patients and modify the management to match the patient’s circumstances thus providing holistic patient care. However, when the narrative does support time learning and working in rural settings, this is often with the caveat that this is only for a short time. The idea that rural medical practice is only a steppingstone to metropolitan practice, subtly suggests that rural communities are suitable only for learners, less experienced doctors or those waiting to find an opportunity to work in a metropolitan area, illustrating how GN facilitates rural exploitation (Fors, 2018).

Healthcare resourcing highlights a systemic bias to ‘centralisation’ where metropolitan areas receive most of the funding and infrastructure development, even after adjusting for population size. This allocation strategy, often justified through economic rationale, neglects the healthcare needs of rural populations. As a result, rural areas with inadequate facilities, and limited access to specialist services, can be less attractive to potential rural doctors. Such disparities in resource distribution not only undermine the quality of care in rural communities but also exacerbates healthcare inequities. Whilst it is understood that access to the medical practitioner is just one of many barriers for rural people to access the healthcare they need, at the time they need, it is a key barrier (Levesque et al., 2013).

Although this study was undertaken in Australia, it is likely that GN will be found in many medical education and training programs around the world and contribute to rural medical workforce shortages internationally. The World Health Organization (WHO) recognises the importance of medical education and training in rural areas and having students experience multiple rural settings, ensuring rural health is positively embedded in healthcare education and that more healthcare students are from rural backgrounds to grow the rural healthcare workforce (World Health Organization, 2021). In addition, the WHO recommends investment in rural infrastructure and rural specific healthcare resources, career development, advancement opportunities and professional networks to be based in rural areas (World Health Organization, 2021), These recommendations while not developed to address GN specifically, are likely to minimise the experiences and potential negative influence of GN during medical education and training.

### Strengths and limitations

The strengths of this study lie in the participants’ breadth of rural and metropolitan clinical learning or working experiences, ensuring a diversity of experiences and robustness of the findings. The use of robust methodology including qualitative method, a constructivist approach facilitated the gathering of rich data, and the RTA meant a systematic and thorough exploration and identification of GN experiences. However, using a qualitative method, may introduce bias from attracting participants with strong feelings on the topic.

A potential limitation of the study is that the term of ‘geographical narcissism’ is not well known and despite providing a definition during the interview, there may have been inadequate time for participants to wholly reflect on their experiences of GN once fully informed of its meaning. However, this may also be a strength of the study as prior understanding of this bias may have complicated self-reflection. Removing prior awareness may have minimised the risk of overthinking and second-guessing one’s responses, as well as being cautious about appearing biased to the interviewer and thus skew authenticity of reflections.

The use of one medical school may not be reflective of GN experiences in other medical schools, however the inclusion of prevocational doctors who attended a variety of medical schools provided balance to the experience of GN during medical school.

## Conclusion

This study has verified that GN is experienced across the continuum of medical school and during prevocational years, during formal teaching, in the clinical environment, during career advice and in the structure of the health system. Whilst reassuring that GN was not often experienced overtly, it is a bias that forms part of the hidden curriculum in medicine. Hearsay, assumptions, and misconceptions appear to foster GN, rather than firsthand experience of working in rural medicine. Building on this new evidence, the impact of GN experiences on medical career choices and workplace locations needs to be understood.

If medical educators, doctors, healthcare leaders and policy makers are unaware or choose not to address GN, its likely contribution to the cycle of inequality will persist. Rural populations will continue to suffer from poorer health outcomes than their metropolitan counterparts, in part due to Geographical Narcissism.

## Electronic supplementary material

Below is the link to the electronic supplementary material.

Supplementary Material 1

## Data Availability

No datasets were generated or analysed during the current study.
