# Supplementary material for: Identifying the experience of geographical narcissism during medical education and training
Source: Adv Health Sci Educ Theory Pract. 2025 May 10;31(1):125–43. doi: 10.1007/s10459-025-10440-9 (PMC12929290; doi:10.1007/s10459-025-10440-9)
Supplement: Supplementary file 1 — Supplementary Material 1 [file 10459_2025_10440_MOESM1_ESM.pdf]

Semi-Structured Interview Questions
for Medical Student Participants

The interviewer will inform participants that they are free to not answer any question without prejudice.

The interviewer will inform participants that the term rural in these interviews means any locations of population <200 000 people, so that we have a shared understanding of rural.

Demographic questions

1. What is your age?
2. What gender do you identify as?
3. Do you identify as an Aboriginal or Torres Strait Islander?
4. Where did you spend the majority of your childhood? Would you class this as a rural or metropolitan area?
5. Are you a domestic or international medical student? If domestic, are you a bonded medical student?
6. What year of Medicine are you in?
7. Where are you currently based?
8. If Y4 – where were you based last year?
9. Do you have a current career plan – GP/Rural Generalist or a non-GP Specialist?
10. What workplace locations interest you for your internship, prevocational doctor years and longer term?

Part A
This group of questions is related to your experience and perception of rural medicine and rural communities during your teaching and learning experiences so far in your medical program.

1. Please describe your rural medical education experiences so far during your medical program.
2. Please describe when and how rural medicine has been taught, discussed, and referred to during the medical program so far?
3. Thinking back, do you recall hearing metropolitan-based, or tutors ever refer to rural medicine, rural doctors, or rural lifestyle as second rate, inferior or criticised in some way?
4. In your opinion, was what you learnt so far in medical school about rural communities, rural medicine, and rural doctors, an accurate reflection of your actual experience or own opinions?

Part B
These questions relate to your likely future medical career workplace locations and interest in rural medicine?

1. Where do you hope to be working for your internship and during your prevocational doctor years?
2. How do you perceive a rural medical career compared to a metropolitan career for yourself?
3. Would you say that you have already ruled in or ruled out working in a rural area at some stage in the future?

Part C
These questions relate to the phenomena of Geographical Narcissism.

1. What do you understand of the term of Geographical Narcissism (GN)?

   Following definition of geographical narcissism to be provided:

Geographical narcissism refers to the either conscious or unconscious bias by metropolitan based individuals, organisations or institutions and their communications and programs, that erodes the quality and value of 'rural' across its workforce, professional standards, lifestyle, and all other facets of society. There is no definitive definition in the literature for geographical narcissism. Effectively, geographical narcissism refers to the concept that everything in larger communities is superior to what exists in smaller communities and propagates the stigma of a minority, in this case rural medicine. GN in medicine implies medical care, services, education, and training provided in other (mostly rural) geographic areas cannot be as good as those provided in metropolitan locations.

2. Now that we have talked a bit about GN, reflecting back on your medical education so far, Where and When during your medical education and training did you experience GN?

3. Thinking about GN experiences in society more broadly, (that is outside of medical education and training), how has it influenced your perception, interest or decisions around rural education, training, work or living?

4. Do you think your rural/metropolitan background, your rural or metropolitan learning experiences have influenced the impact of GN on your interest in being a rural intern, junior doctor or after you gain your fellowship?

Semi-Structured Interview Questions
for Pre-Vocational Doctor Participants

The interviewer will inform participants that they are free to not answer any question without prejudice.

The interviewer will inform participants that the term rural in these interviews means any locations of population <200 000 people, so that we have a shared understanding of rural.

Demographic questions

1. What is your age?
2. What gender do you identify as?
3. Do you identify as an Aboriginal or Torres Strait Islander?
4. Where did you spend the majority of your childhood? Would you class this as a rural or metropolitan area?
5. Are you a domestic or international medical graduate? If domestic, were you a bonded medical student?
6. At which university did you gain your medical degree?
7. What year did you graduate?
8. Where are you currently working?
9. If PGY2 or more, where did you work for your internship and the PVJD years up to now.
10. Do you have a current career plan – GP, Rural Generalist or a non-GP Specialist?
11. What workplace locations interest you for your prevocational junior doctor years and longer term?

Part A

This group of questions is related to your experience and perception of rural medicine and rural communities during your teaching and learning experiences as a MEDICAL STUDENT.

1. Please describe your rural medical education experiences during your medical degree.
2. Please describe what you remember about how rural medicine was taught, discussed, or referred to when you were a medical student, including during clinical placements?
3. Thinking back, do you recall hearing metropolitan-based doctors or tutors ever refer to rural medicine, rural doctors, or rural lifestyle as second rate, inferior or criticised in some way?
4. In your opinion, was what you learnt in medical school about rural communities, rural medicine, and rural doctors an accurate reflection of your actual experience or own opinions?

Part B

These questions relate to your experience and perception of rural medicine and rural communities during any of your workplace experiences as a JUNIOR DOCTOR.

1. Do you recall ever hearing metropolitan doctors in the workplace refer to rural medicine, rural doctors, and/or rural lifestyle as second rate, substandard or criticised in some way?
2. Have you ever heard a metropolitan doctor advise you or your colleagues that working in a rural hospital or health care facility will affect medical career development?

Part C

These questions relate to your likely future medical career workplace locations and interest in rural medicine?

1. Where do you think you will be working for your future Prevocational doctor years?

2. How do you perceive a rural medical career compared to a metropolitan career for yourself?
3. Would you say that you have already ruled in or ruled out working in a rural area at some stage in the future?

Part D
These questions relate to the phenomena of Geographical Narcissism.

1. What do you understand of the term of Geographical Narcissism?

   Following definition of geographical narcissism to be provided:

   Geographical narcissism refers to the either conscious or unconscious bias by metropolitan based individuals, organisations or institutions and their communications and programs, that erodes the quality and value of 'rural' across its workforce, professional standards, lifestyle, and all other facets of society. There is no definitive definition in the literature for geographical narcissism. Effectively, geographical narcissism refers to the concept that everything in larger communities is superior to what exists in smaller communities and propagates the stigma of a minority, in this case rural medicine. GN in medicine implies medical care, services, education, and training provided in other (mostly rural) geographic areas cannot be as good as those provided in metropolitan locations.
2. Now that we have talked a bit about GN, reflecting back on your medical education and training so far, Where and When did you experience GN?
3. Thinking about GN experiences in society more broadly, (that is outside of medical education and training and the workplace), how has it influenced your perception, interest or decisions around rural education, training, work or living?
4. Do you think your rural/metropolitan background, or your rural/metropolitan learning experiences has influenced the impact of GN experiences on your interest in being a rural junior doctor or to work rurally after you gain your fellowship?
